# Direct observation of dynamic interaction between a functional group in a single SBR chain and an inorganic matter surface

**DOI:** 10.1038/s41598-018-32382-6

**Published:** 2018-09-18

**Authors:** Ken-ichi Shinohara, Yuu Makida

**Affiliations:** School of Materials Science, Japan Advanced Institute of Science and Technology (JAIST), 1-1 Asahi-dai, Nomi, Ishikawa 923-1292 Japan

## Abstract

As a composite of hybrid organic-inorganic materials, blending hydrophilic silica microparticles with oil-extended rubber can improve vehicle tire performance but the nanometer scale effects of microparticle inclusion have not been thoroughly studied. Here, we used atomic force microscopy (AFM) video imaging to closely investigate the behavior of functionalized and unmodified styrene-butadiene rubber (SBR), as models for tire rubber, on mica surfaces. The hydrophilic silica microparticle surface could be simulated by a mica substrate because both have silanol groups on their surface. Using AFM video imaging, we tracked the behavior of individual SBR polymer chains on mica surfaces to reveal how polymer modification affects the interaction of SBR with mica surfaces. We measured the diffusion coefficients and spring constants of single SBR polymer chains for the first time, demonstrating that it is possible to parameterize the relationship between the molecular dynamic structure of a polymer and rubber properties of the vulcanized compound.

## Introduction

**Figure 1:**
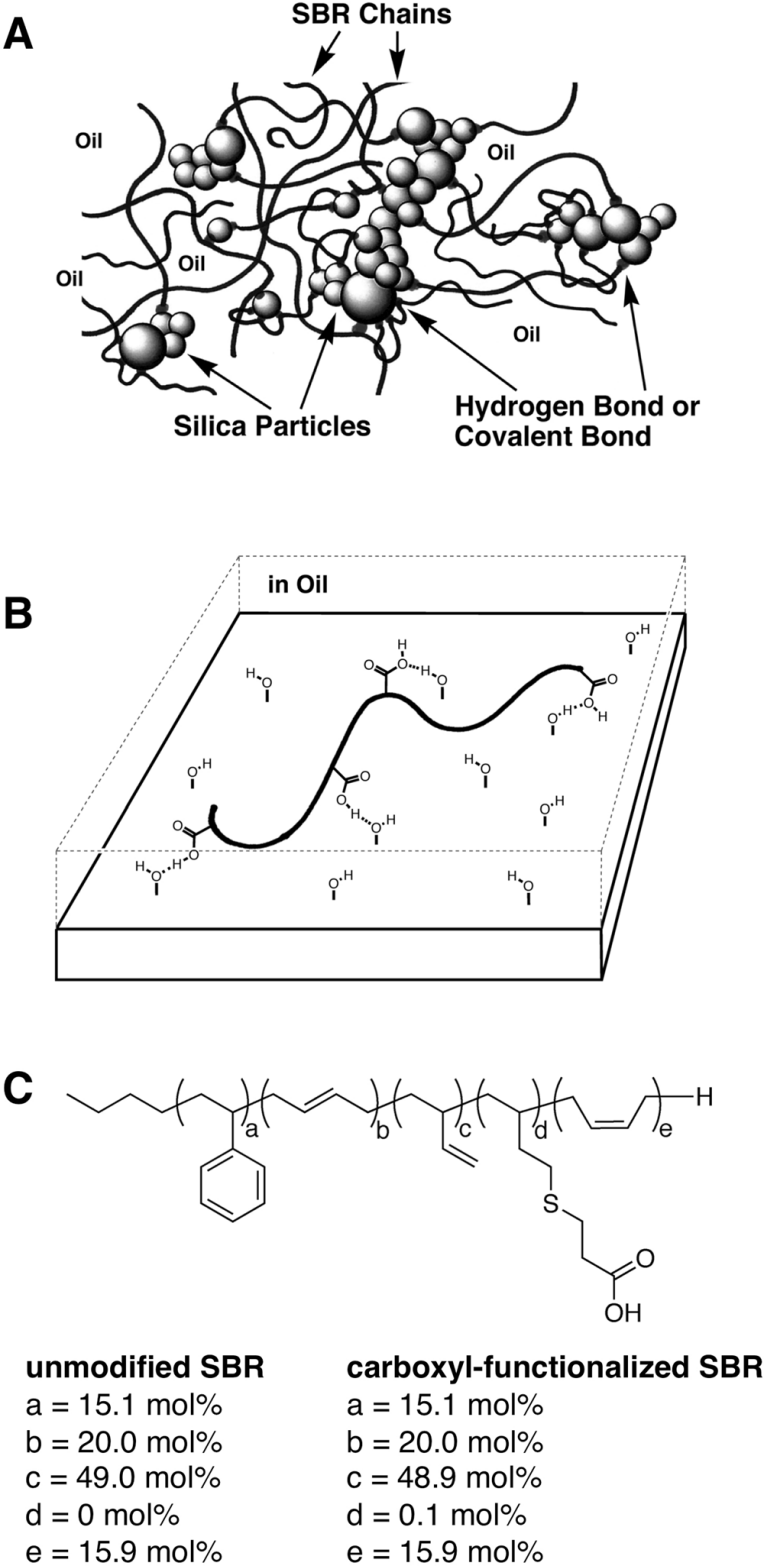
Schematic illustrations of (**A**) hydrogen bonding between the functionalized styrene-butadiene rubber (SBR) polymer chains and hydrophilic silica microparticles in oil-extended rubber for tires, and (**B**) hydrogen bonding between a carboxyl-functionalized SBR chain and silanol groups on a mica substrate in an oil. The hydrophilic silica microparticle surface can be simulated by a mica substrate. (**C**) Chemical structure of unmodified and carboxyl-functionalized SBR.

A composite of hybrid organic-inorganic materials display useful properties. In order to understand the hybrid materials, a study of the dynamics at organic-inorganic interface in a nanometer scale is important. As a representative, styrene-butadiene rubber (SBR) is used to determine the characteristic features of car tires^1–7^. Blending hydrophilic silica microparticles with oil-extended rubber to form a hybrid organic-inorganic material can remarkably improve tire performance^8^. Tire performance factors that could be further improved include wet-grip performance and fuel consumption. Modifying target positions in the SBR chain to facilitate bonding interactions with hydrophilic silica microparticles can regulate the molecular motion of the SBR polymer chain to produce tires with improved performance, such as lower exothermicity and rolling resistance (Fig. 1A)^9–11^. Actually, the wet-grip performance of a vulcanized compound of a silica-blended a carboxyl-functionalized SBR improved and the rolling resistance decreased, compared with that of a silica-blended unmodified SBR^12^.

If we can analyze the structure and dynamics of a functionalized SBR polymer chain^13–15^, we can not only determine the position of the functional group introduced into the polymer chain, but also parameterize the degree of bonding/interaction of the functional group with silica microparticles. The hydrophilic silica microparticle surface can be simulated by a mica substrate^16^, because both have silanol groups on their surface. This makes it possible to determine the position of functional group introduced into the functionalized SBR polymer chain and the bonding of these groups to the hydrophilic silica microparticles, which will provide parameters for the design of high-performance silica-blended tires.

In this work, to clarify the relationship between the structure and rubber properties of functionalized SBR, we directly analyze the intramolecular position and properties of the functional groups in modified SBR by atomic force microscopy (AFM) video imaging. AFM video imaging can directly observe the motion of a single polymer chain on a surface^17,18^. Such direct observation of a polymer chain deepens our understanding of its structure and function^19^. Using AFM video imaging, the structure and dynamics of single polymer chains of functionalized and unmodified SBR are determined. We also quantitatively evaluate the interaction of an SBR polymer chain with a mica substrate in an organic medium (Fig. 1B).

## Method

According to the previously reported method^12,20^, an unmodified SBR and a carboxyl-functionalized SBR (Fig. 1C and Scheme S1) were synthesized. A dilute tetrahydrofuran (THF) solution (<1 × 10^−6^ *w*/*v*) of unmodified SBR (*M*_w_: 1.54 × 10^5^, *M*_w_/*M*_n_: 1.02) and carboxyl-functionalized SBR (SBR-(COOH)-: *M*_w_: 2.14 × 10^5^, *M*_w_/*M*_n_: 1.17) was prepared, respectively. Dehydrated THF (Kanto Chemical, Tokyo, Japan) was used to prevent aggregation of the SBR polymer chains. A freshly cleaved mica surface of the muscovite substrate (Nilaco, Tokyo, Japan) was obtained using adhesive tape, and any adsorbed water on the mica surface was removed by rinsing with dehydrated THF in a dry air atmosphere (RH < 25%). The samples were prepared by casting the dilute THF solution of the polymer (1 μL) onto a mica substrate. The substrate was rinsed with THF (1.0 mL) after standing for ca. 20 s to remove excess polymer chains and leave isolated chains on the substrate. As another method, a sample was prepared by spin-casting (1,500 rpm) the dilute polymer solution (1 μL) onto a mica substrate in a dry air atmosphere. Through the bonding/interaction of the exposed hydroxyl groups on the mica surface with the functional groups in the SBR polymer chains, each single chain of the functionalized SBR polymer was dispersed as shown in Fig. 1B and adsorbed onto the mica surface in a suitably stretched state. If the dilute solution of the polymer is cast/dried on the substrate, the polymer chains aggregate and form globules easily, so the above technique is necessary. We modified the specifications of a fast-scanning atomic force microscope (NVB500, Olympus, Tokyo, Japan) in dynamic (tapping) mode to observe isolated polymer chains using a cantilever (AC-10EGS, Olympus, Japan or USC-F1.2-k0.15, Nano World AG, Switzerland)^13,14^. A fast-scanning (high-speed) atomic force microscopy (AFM) video imaging of the structural dynamics in a single polymer chain was captured at 25 ± 1 °C in an organic solvent. We confirmed that *n*-octylbenzene (Tokyo Chemical Industry (TCI), Tokyo, Japan), *n*-octylether (TCI), hexadecane (Wako Chemical, Tokyo, Japan), and decamethyltetrasiloxane (DMTS; TCI) are useful as observation solvents for AFM video imaging of single chains. Because the interaction between the polymer chain and a mica substrate also depends on the affinity to observation solvent, a solvent suitable for observing the dynamic state of the polymer chain was selected. The structural dynamics of the unmodified SBR polymer were observed in DMTS by AFM video imaging after the process of adsorption on a mica substrate. On the other hand, *n*-octylbenzene was used as a solvent for AFM video imaging of carboxyl-functionalized SBR. In order to discuss the affinity of solvents, we used the solubility parameter^21^. Calculated solubility parameters in SBR (styrene content = 25 wt%), *n*-octylbenzene, and DMTS were 18.2, 18.1, and 14.3 (J/cm^3^)^1/2^, respectively.

Here, the hydrophilic silica microparticle surface can be simulated by a mica substrate, because both have hydroxyl groups (as silanol) on their surface. The densities of surface hydroxyl groups of mica (muscovite) and hydrophilic silica microparticles were 3.2 and 4.7 per nm^2^, respectively.

In the analysis of dynamics in a polymer chain, each measurement point of the video-imaged polymer chain was tracked and the mean square displacement (MSD) for a certain time Δ*t* was plotted against Δ*t*,

$$MSD({\rm{\Delta }}t)=\overline{{[{\rm{\Delta }}x({\rm{\Delta }}t)]}^{2}+{[{\rm{\Delta }}y({\rm{\Delta }}t)]}^{2}}.$$

The diffusion coefficient *D* (nm^2^/s) was calculated by dividing the slope of the linearly approximating the MSD-Δ*t* plots of each measurement point by four. These tracking points were determined by the numbering at equal intervals along the single chain trunk at each frame of the AFM movie. The spring constant of a single SBR chain was also calculated, see the supplementary information for detail.

Molecular modeling was conducted by all-atom molecular dynamics (MD) for a carboxyl-functionalized SBR in *n*-octylbenzene, see the supplementary information for detail.

## Results and Discussion

### An unmodified SBR chain on mica in oil

**Figure 2:**
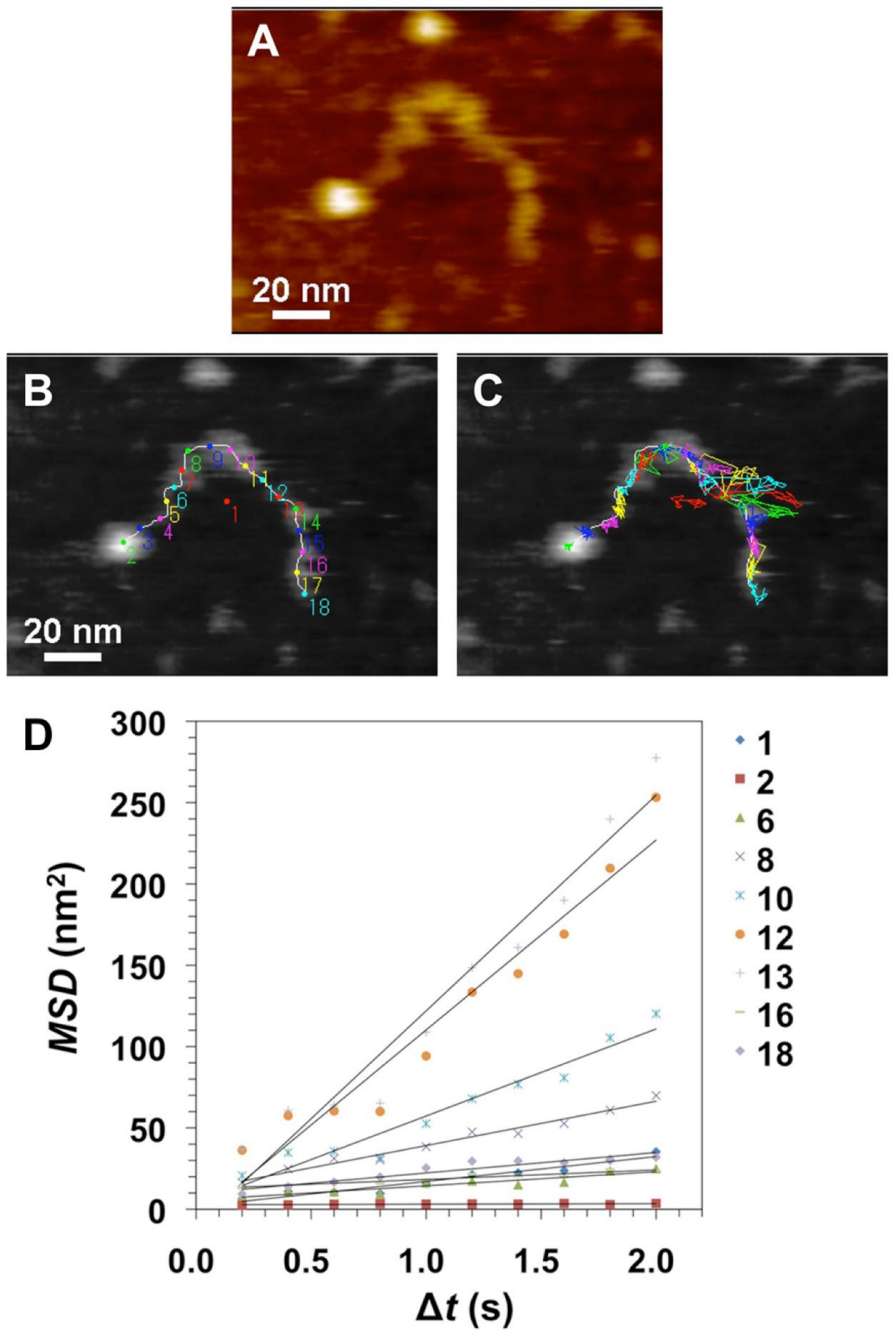
Dynamic structural analysis of an unmodified styrene-butadiene rubber (SBR) polymer chain on mica under decamethyltetrasiloxane (DMTS) at 25 ± 1 °C. (**A**) A snapshot in an atomic force microscopy (AFM) video (Movie S2) of a single chain. (**B**) The measurement points were indicated over an AFM snapshot. (**C**) Trajectories of the points in (**B**). (Movie S3) Scale bar: 20 nm, Z: 7.2 nm. Frame rate: 5.0 fps. (**D**) Mean square displacement (*MSD*)–Δ*t* plots for the points.

**Figure 3:**
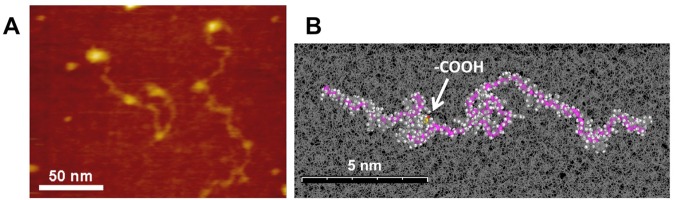
(**A**) Single-molecule imaging of the structure of two isolated polymer chains of carboxyl-functionalized styrene-butadiene rubber (SBR) on mica under *n*-octylbenzene at 25 ± 1 °C (Movie S5). Snapshot AFM image of a fast-scanning atomic force microscopy (AFM) movie; X: 200 nm, Y: 150 nm, Z: 7.2 nm. Rate: 5.0 fps. (**B**) A snapshot of all-atom MD simulated structure of a single chain of carboxyl-functionalized SBR (CPK model) in *n*-octylbenzene as a solvent. Dynamic globular (ball-like) structures were formed partially in a SBR chain. The position of carboxyl group was indicated by an arrow. The backbone was displayed in purple. Solvent molecules are indicated by line model and hydrogen atoms are omitted for simplified to view.

An AFM video imaging of a macromolecular motion in single chains of unmodified SBR on a mica surface was performed in decamethyltetrasiloxane (DMTS) at 25 ± 1 °C (Movie S1). Structural dynamics of several single polymer chains were observed. The height of the polymer chain was 1.09 ± 0.22 nm (Fig. S1A). Based on the size of the molecular model, it was considered that the observed string-like structure was the single chains. In order to discuss the dynamic interaction between a single chain and an inorganic substrate, the isolated polymer chain of unmodified SBR was also observed (Movie S2). A snapshot of AFM image from the movie was shown in Fig. 2A. As shown in Fig. 2B, measurement points for displacement were set at equal intervals along the trunk of the single chain [a chain end to the other chain end (2–18)], and also set at the chain centroid (1) (Movie S3). The mobility of each point determined based on the trajectory data for the single chain was measured by AFM video imaging. The trajectories of each tracking point were also shown (Fig. 2C). Figure 2D displays the mobility of the measuring points in terms of their mean square displacement (MSD) over time (Δ*t*) based on the trajectory data in Fig. 2C. The MSD plots were fitted by the linear regression lines. The diffusion coefficient (*D*) values determined for an unmodified SBR polymer chain are presented in Table S1. The *D* values were wide ranging from 0.1 to 33 nm^2^/s, indicating that the dynamic interaction between segments in an unmodified SBR chain and a mica was diverse. *D*_18_ (3.2 nm^2^/s) at a chain-end was 32 times higher than *D*_2_ (0.1 nm^2^/s) at an another chain-end formed a ball-like structure of 2.5 nm height (Fig. S1B). *D*_13_ (33 nm^2^/s) in the middle of the chain with high mobility was 10 times higher than *D*_18_ at the chain-end. This high mobility was caused by the flexibility of unmodified SBR chain. The structural dynamics differs from that for the carboxyl-functionalized SBR chain as shown in the next part (Fig. 3 and Table S1). The movement of the centroid *D*_1_ (3.8 nm^2^/s) in the unmodified SBR chain was enabled in the absence of the functional groups as anchoring points in a polymer chain.

The AFM movie revealed that observation points 10 to 14 (Fig. 2B,C) displayed marked stretching that was considered as a characteristic of rubber (Movie S3). Dynamic structure as partially coil spring is considered to have been formed in the SBR chain. Actually, this consideration is supported as dynamic structures with a height of ca. 1 nm are observed in the chain. The stretching behavior was measured by the trajectories (100 points) along a polymer chain (Fig. S2 and Movie S4). As the result, the average length of the unmodified SBR polymer chains was 140 nm with a standard deviation (SD) of 17.2 nm (*n* = 30). Based on the SD, a spring constant (*k*_chain_) was calculated as 2.78 × 10^−2^ pN/nm (supplementary information). That is, the SBR chain behaves as a molecular spring^22^ that moves driven by thermal fluctuation.

### A carboxyl-functionalized SBR chain on mica in oil

**Figure 4:**
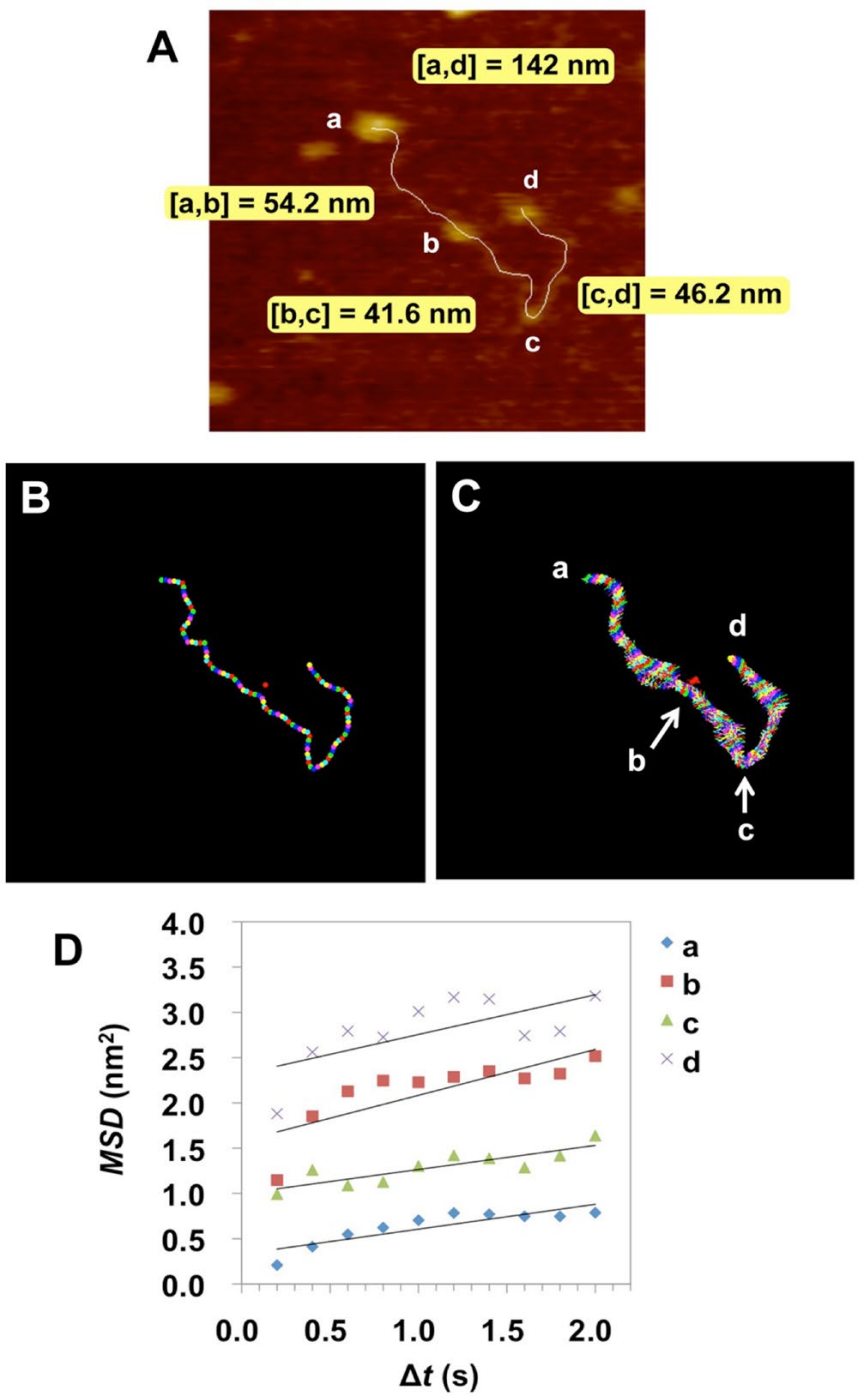
(**A**) Functionalized positions (a–d) in a single polymer chain of carboxyl-functionalized styrene-butadiene rubber (SBR) on mica under *n*-octylbenzene at 25 ± 1 °C. This image is a snapshot from an atomic force microscopy video. XY: 139 nm × 139 nm, Z: 7.2 nm. Frame rate: 5.0 fps (Movie S6). (**B**–**D**) Dynamic structural analysis of a functionalized SBR chain. (**B**) Snapshot of a single chain with 101 measurement points. (**C**) Trajectories of the 101 points in (**B**). An isolated point (red) was the centroid of the chain. (**D**) Mean square displacement (*MSD*)-Δ*t* plots for the anchoring points (a–d) in (**A**,**C**).

After living anionic polymerization of styrene and butadiene, functional groups were introduced by the thiol-ene click reaction (Scheme S1). Since four equivalents of 3-mercaptopropionic acid to the living anionic polymerization initiator was added to the reaction system, it was calculated that an average of four carboxyl groups per a chain was introduced. An AFM video of a carboxyl-functionalized SBR polymer chain were shown in Movie S5 and Fig. 3A (snapshot). The height of the polymer chain was 0.55 ± 0.17 nm. Based on the size of the molecular model (Fig. 3B), it was considered that the observed string-like structure was a single SBR chain. Ball-like structures with a height of 1–2 nm were identified at four sites in the polymer chain (a–d in Fig. 4) (Fig. S3). These four structures were fixed, and thus represent the positions of introduced carboxyl groups that are linked to the mica surface by hydrogen bonds.

Because the modifying group has high polarity, in the organic solvent the neighboring SBR segment covers the surface, so a ball-like structure is likely to form. Approximately four similar ball-like structures were observed in other molecular chains (Movie S5). This tendency is more conspicuous in DMTS, which has a large ΔSP (difference of two solubility parameters) value (Table S1), and the ball-like structures with larger size were confirmed in Movie S7. In contrast, unmodified SBR contained fewer ball-like structures even in DMTS (Movies S1 and S2). These ball-like structures were considered to be globules locally formed in the chain due to the high polarity of carboxyl group. By all-atom MD simulation, a ball-like structure around the carboxyl group has been confirmed (Fig. 3B).

The length of line segments between the four anchoring points (a–d) were measured as shown in Fig. 4A. A white line indicates the trunk of the polymer chain. The anchoring positions of a and d were located near the chain ends. The length between anchoring points was 54.2 nm between a and b, 41.6 nm between b and c, and 46.2 nm between c and d. Based on the 100-points measurement of the trajectories along a polymer chain (Fig. 4B,C), the average length of the carboxyl-functionalized SBR polymer chains was 142 nm with SD of 2.84 nm (*n* = 31). Chain length change of the functionalized SBR over time was smaller than unmodified SBR (17.2 nm), confirming the strong anchoring ability of the carboxyl group. Based on the SD, a spring constant (*k*_chain_) was calculated as 1.02 pN/nm (supplementary information). This value is 36.7 times higher than the spring constant of the unmodified SBR chain. The effect of modifying groups could be evaluated by spring constant.

The 100 points obtained by equally dividing the trunk, and the centroid were shown in Fig. 4B. These points were from a typical snapshot image in the AFM movie (Movie S6). The trajectories of each point were also shown (Fig. 4C). Briefly, the segment with narrow trajectory shows low mobility. The points a, b, c and d in Fig. 4C were confirmed to be bonding the mica substrate because the trajectory width was remarkably narrow. In order to measure the diffusion coefficient (*D*), the MSD-Δ*t* plots based on the trajectories for four points (a–d) were indicated in Fig. 4D. Since the *D*_a_, *D*_b_, *D*_c_ and *D*_d_ were measured to 0.1 nm^2^/s, it was also confirmed that this polymer chain was fixed at these four points. Thus, *D* could be used to determine the anchoring ability to bind to the mica surface.

### Network of carboxyl-functionalized SBR chains on mica in oil

**Figure 5:**
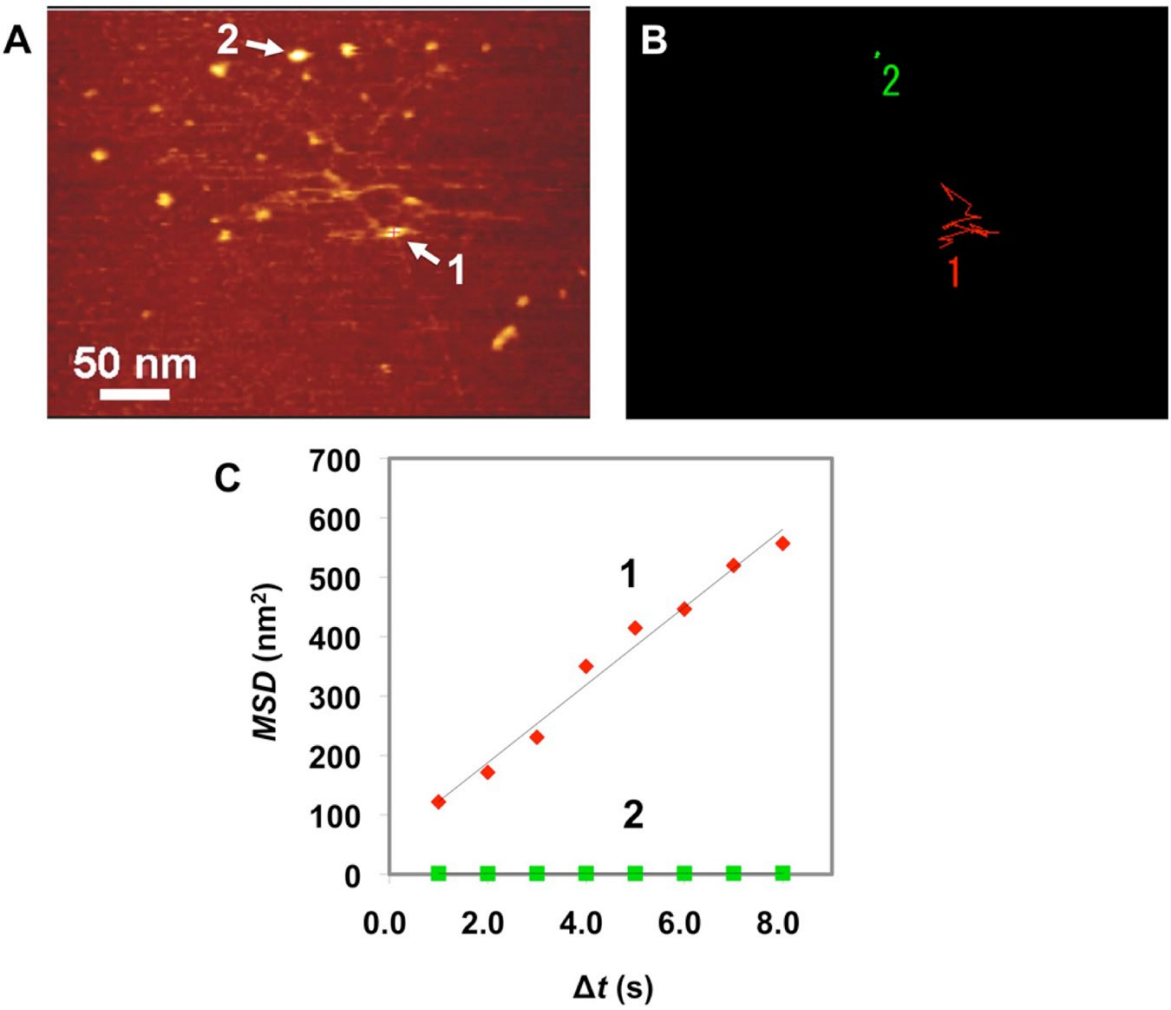
Dynamic structural analysis of a polymer network of a functionalized styrene-butadiene rubber (SBR). (**A**) Snapshot from an atomic force microscopy video (Movie S8) of a network consisting of a few chains of carboxyl-functionalized SBR on mica under *n*-octylbenzene at 25 ± 1 °C. (**B**) Trajectories of an entangled point 1 (red line) and an anchoring point 2 (green line). (**C**) Mean square displacement (*MSD*)–Δ*t* plots of points 1 and 2 in (A).

In order to observe the network dynamics of polymer chains, an entangled structure consisting of a few carboxyl-functionalized SBR polymer chains on a mica substrate in *n*-octylbenzene at 25 ± 1 °C was then investigated (Fig. 5 and Movie S8). A tracking point 1 in Fig. 5A indicated a site having high mobility where a few polymer chains form the dynamic entangling structure, while point 2 is an anchoring point where a modified group strongly bonds to the mica substrate via hydrogen bonding, the trajectories were shown in Fig. 5B. The MSD-Δ*t* plots (Fig. 5C) were used to determine *D* values. We found that *D*_1_ and *D*_2_ were 16.4 and 1.66 × 10^−2^ nm^2^/s, respectively. Thus, *D* of the dynamically entangled structure is ca. 1,000 times higher than that of the anchoring point. The dynamic entangling structure is composed of a few polymer chains that expand and shrink randomly upon receiving the tension generated from the surrounding anchoring points by thermal fluctuation (Fig. S8). Even in the network structure, it was directly confirmed that the functional group bonded to the mica surface. This is the first direct observation of the origin of rubber elasticity from a small number of the entangled polymer chains having the anchoring segments, and the cooperative effect of polymer chains (Movie S9).

By climbing up the molecular-level hierarchy from a single polymer chain to a few chains, we can get closer to understanding the essential nature of a material. It is therefore possible to gauge the molecular level at which “polymer-like” and “materials-like” nature is expressed.

## Conclusion

In this study, we focused on the combination of SBR-mica with oil as a hybrid organic-inorganic material. The macromolecular movement of the functionalized SBR in an organic medium on mica is considered to resemble that of an actual functionalized SBR interacting with silica in a tire compounds. Therefore, observing and quantifying such behavior should reveal features of tire performance from molecular level, such as energy loss and wet grip. The wet grip performance (tan δ at 0 °C) of a vulcanized compound of a silica-blended functionalized SBR was 1.4 times higher than that of a silica-blended unmodified SBR^12^. This result strongly correlates with the diffusion coefficient of a single polymer chain (Table S1). Although the results for carboxyl-functionalized SBR were presented here, by analyzing the dynamics of various functionalized SBRs, quantification and ranking of the fixing ability of functional groups may be achieved, and development guidelines can be clarified for other polymers used in tires. In other words, it is possible to parameterize the correlation between the polymer structure and function of rubber compounds, such as the type of polymer structure able to be fixed to the filler silica surface. Understanding will be deepened further by correlation with the tire performance index (tan δ) and results of X-ray analysis and computer simulation^23,24^.

Finally, this study method of quantification of a dynamic interaction between a polymer chain and an inorganic matter can be applied not only to SBR tire composite but also to research on various soft matters from hybrid organic-inorganic materials.

## Electronic supplementary material

Supplementary Information

Movie S1

Movie S2

Movie S3

Movie S4

Movie S5

Movie S6

Movie S7

Movie S8

Movie S9
