# Supplementary material for: Direct observation of dynamic interaction between a functional group in a single SBR chain and an inorganic matter surface
Source: Sci Rep. 2018 Sep 18;8:13982. doi: 10.1038/s41598-018-32382-6 (PMC6143511; doi:10.1038/s41598-018-32382-6)
Supplement: Supplementary file 1 — Supplementary Information [file 41598_2018_32382_MOESM1_ESM.pdf]

# Supplementary Information for

## Direct observation of dynamic interaction between a functional group in a single SBR chain and an inorganic matter surface

Ken-ichi Shinohara* and Yuu Makida

correspondence to: shinoken@jaist.ac.jp

**Contents:**

**Materials and methods**

**Synthesis of a carboxyl-functionalized SBR**

**AFM video imaging and the analytical method**

**Molecular modeling by all-atom MD**

**Results and discussion**

**Spring sonstant of a single polymer chain**

**Relationships between tire performance and single polymer chains**

**Captions:**

**Chart S1.** Chemical structure of unmodified and carboxyl-functionalized SBR

**Chart S2.** Fast-scanning AFM head with a fluid cell

**Chart S3.** Classical spring model of a carboxyl-functionalized SBR

**Scheme S1.** thiol-ene click reaction to introduce a carboxyl group into a SBR chain

**Table S1.** Diffusion coefficients of a single SBR chain

**Fig. S1.** Line profile of AFM image of single polymer chain of unmodified SBR

**Fig. S2.** Chain length-time plots of an unmodified SBR

**Fig. S3.** Line profile of AFM image of single polymer chain of a carboxyl-functionalized SBR

**Fig. S4.** Chain length-time plots of a carboxyl-functionalized SBR

**Fig. S5.** Dynamic structural analysis of a carboxyl-functionalized SBR on mica under *n*-octylbenzene at 25 ± 1 °C. (A) The 18-points measurement points were indicated over an AFM snapshot. (B) The trajectories. (C) MSD-Δ*t* plots in the single polymer chain

**Fig. S6.** MSD-Δ*t* plots (Δ*t* ≤ 0.8 s from Fig. S5C) of 18-points measurement in single polymer chain of a carboxyl-functionalized SBR for calculation of the *D* values as shown in Table S1

**Fig. S7.** All-atom molecular dynamics (MD) simulated structure of a 100-mer carboxyl SBR chain in *n*-octylbenzene

**AFM video:**

**Movie S1.** AFM video imaging of the structural dynamics of unmodified SBR in decamethyltetrasiloxane (DMTS) at 25 ± 1 °C. Fast-scanning AFM image; X: 300 nm, Y: 225 nm, Z: 7.2 nm. Frame rate: 5.0 fps.

**Movie S2.** AFM video imaging of the structural dynamics of a single polymer chain of unmodified SBR on a mica substrate in DMTS at 25 ± 1 °C (Fig. 2A). Fast-scanning AFM image; X: 150 nm, Y: 113 nm, Z: 7.2 nm. Frame rate: 5.0 fps.

**Movie S3.** Trajectories of single polymer chain of unmodified SBR (Fig. 2B)

**Movie S4.** AFM video imaging and the 101-points measurement of the trajectories along a polymer chain of unmodified SBR on a mica substrate under DMTS at 25 ± 1 °C (Fig. 2A). Fast-scanning AFM image; X: 150 nm, Y: 113 nm, Z: 7.2 nm. Frame rate: 5.0 fps.

**Movie S5.** AFM video imaging of the structural dynamics of two isolated polymer chains of carboxyl-functionalized SBR on a mica substrate under *n*-octylbenzene at 25 ± 1 °C (Fig. 3). Fast-scanning AFM image; X: 200 nm, Y: 150 nm, Z: 7.2 nm. Rate: 5.0 fps.

**Movie S6.** AFM video imaging and the 100-points measurement of the trajectories along a polymer chain of carboxyl-functionalized SBR on a mica substrate under *n*-octylbenzene at 25±1 °C (Fig. 4). Fast-scanning AFM image; X: 139 nm, Y: 139 nm, Z: 7.2 nm. Frame rate: 5.0 fps.

**Movie S7.** Structural dynamics of a few chains of the carboxyl-functionalized SBR on a mica substrate under DMTS at 25 ± 1 °C. Fast-scanning AFM image; X: 150 nm, Y: 113 nm, Z: 7.2 nm. Rate: 10 fps. Video images are panned to observe a wide range.

**Movie S8.** AFM video imaging and trajectory analysis (an entangling structure, point 1 in Fig. 5) of the structural dynamics of a polymer network of a few chains of the carboxyl-functionalized SBR on a mica substrate under *n*-octylbenzene at 25 ± 1 °C. Fast-scanning AFM image; X: 397 nm, Y: 298 nm, Z: 7.2 nm. Frame rate: 1.0 fps.

**Movie S9.** Network dynamics of polymer chains of carboxyl-functionalized SBR with 4-frames averaging process

**Materials and methods**

**Synthesis of unmodified SBR and carboxyl-functionalized SBR**

**Chart S1**

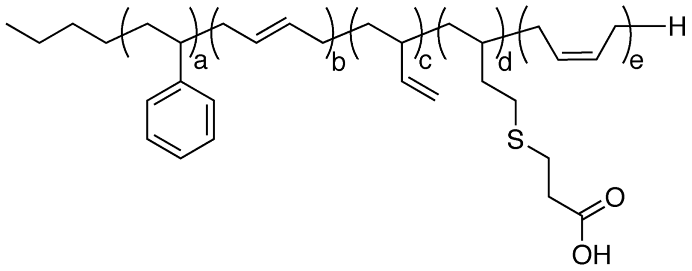

| **unmodified SBR** | **carboxyl-functionalized SBR** |
| --- | --- |
| **a = 15.1 mol%** | **a = 15.1 mol%** |
| **b = 20.0 mol%** | **b = 20.0 mol%** |
| **c = 49.0 mol%** | **c = 48.9 mol%** |
| **d = 0 mol%** | **d = 0.1 mol%** |
| **e = 15.9 mol%** | **e = 15.9 mol%** |

According to a previously reported method,[1,2] unmodified styrene-butadiene rubber (SBR) (Chart S1) was synthesized by a living anionic polymerization reaction of styrene and 1,3-butadiene using *n*-butyllithium as the initiator. After completion of the copolymerization reaction, in order to synthesize carboxyl-functionalized SBR (Chart S1), 3-mercaptopropionic acid (the thiol, **2**) and lauroyl peroxide (radical initiator, **3**) were added under stirring.[3] The product was dried under reduced pressure. Four equivalents of 3-mercaptopropionic acid (**2**) to one equivalent of the living anionic polymerization initiator were added to the reaction system.

The content of the styrene unit in the polymer was determined from the refractive index using the Abbe refractometer (NAR-3T, ATAGO, Tokyo, Japan) according to international standard ISO 2453:1991. The amount of vinyl unit in the polymer was determined from the infrared absorption intensity. The absorption peak at around 911 $cm^{-1}$ is that of the 1,2-vinyl unit, the peak at around 735 $cm^{-1}$ is that of the 1,4-cis unit, and the peak at around 967 $cm^{-1}$ is that of the 1,4-trans unit. Fourier transform infrared spectrometer (FT/IR-470 plus, JASCO, Tokyo, Japan) was used.

Regarding the reaction mechanism (Scheme S1), after hydrogen abstraction from thiol **2** by radical initiator **3**, the resulting thiyl radical **4** was added to vinyl group **1** in the SBR chain. The generated radical **5** abstracted a hydrogen atom from another thiol to form a stable structure (**6**). In each reaction cycle, a thiyl radical (**4**) was generated anew, which attacked another vinyl group, inducing a chain reaction. Because 3-mercaptopropionic acid was used as the thiol (**2**) in this reaction, carboxyl groups were introduced into the polymer chain.

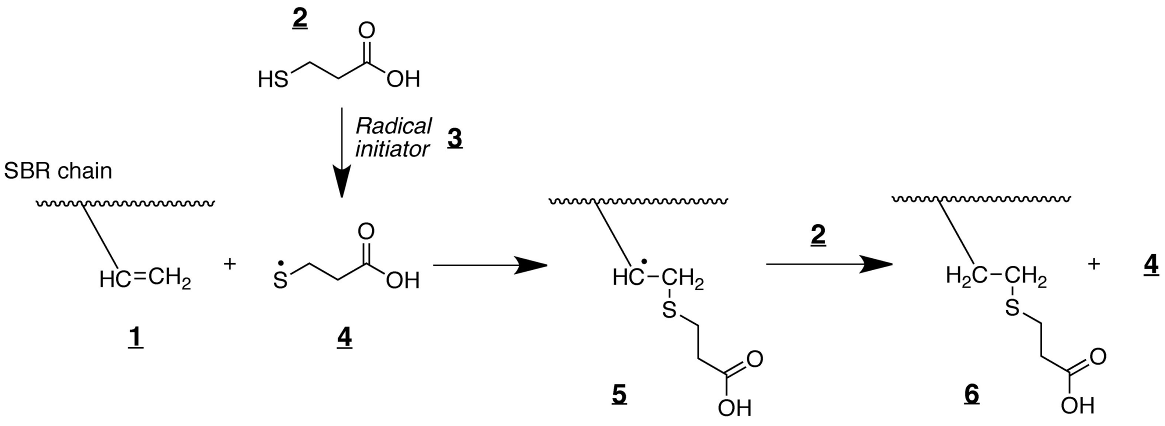


**Scheme S1. Synthesis of a carboxyl-functionalized SBR.**

## AFM video imaging and the analytical method

According to the previously reported method,[1,2] an unmodified SBR and a carboxyl-functionalized SBR (Chart S1 and Scheme S1) were synthesized. A dilute tetrahydrofuran (THF) solution ($< 1 \times 10^{-6}$ *w/v*) of unmodified SBR ($M_w$: $1.54 \times 10^5$, $M_w/M_n$: 1.02) and carboxyl-functionalized SBR (SBR-(COOH)-: $M_w$: $2.14 \times 10^5$, $M_w/M_n$: 1.17) was prepared, respectively. Dehydrated THF (Kanto Chemical, Tokyo, Japan) was used to prevent aggregation of the SBR polymer chain. A freshly cleaved mica surface of the muscovite substrate (Nilaco, Tokyo, Japan) was obtained using adhesive tape, and any adsorbed water on the mica surface was removed by rinsing with dehydrated THF in a dry air atmosphere (RH < 25%). The samples were prepared by casting the dilute THF solution of the polymer (1 μL) onto a mica substrate. The substrate was rinsed with THF (1.0 mL)

after standing for ca. 20 s to remove excess polymer chains and leave isolated chains on the substrate. As another method, a sample was prepared by spin-casting (1,500 rpm) the dilute polymer solution (1 μL) onto a mica substrate in a dry air atmosphere. Through the bonding/interaction of the exposed hydroxyl groups on the mica surface with the functional groups in the SBR polymer chains, each single chain of the functionalized SBR polymer was dispersed as shown in Fig. 1B and adsorbed onto the mica surface in a suitably stretched state. If the dilute solution of the polymer is cast/dried on the substrate, the polymer chains aggregate and form globules easily, so the above technique is necessary.

**Chart S2.**

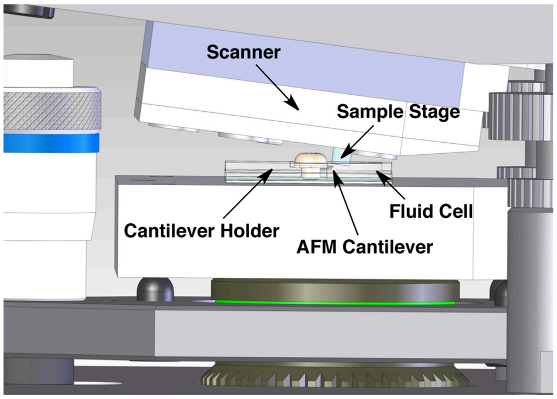


We modified the specifications of a fast-scanning atomic force microscope (NVB500, Olympus, Tokyo, Japan) in dynamic (tapping) mode to observe isolated polymer chains.[4,5] The ultra small cantilever having a low spring constant of around 0.1 N/m and high resonance frequency of over 1 MHz in air was used (AC-10EGS, Olympus, Japan or USC-F1.2-k0.15, Nano World AG, Switzerland). Here, a fast-scanning atomic force microscope (AFM) offers outstanding performance for observing the structural dynamics of single-molecules in aqueous solution.[6,7] However, we modified the AFM in order to use even in the organic solvent, allowing successful imaging of the structural dynamics of a single polymer chain. Chart S2 shows the AFM head with a fluid cell (designed by using the CAD software: SOLIDWORKS, Dassault Systèmes SolidWorks

Corporation, Waltham, MA, USA) for imaging in an organic solvent. The cell volume was 9 µL. AFM video imaging of the structural dynamics of a single polymer chain of the functionalized SBR was captured at 25 ± 1 °C in an organic solvent. We confirmed that *n*-octylbenzene (Tokyo Chemical Industry (TCI), Tokyo, Japan) (Fig. S5, Movie S5), *n*-octylether (TCI), hexadecane (Wako Chemical, Tokyo, Japan), and decamethyltetrasiloxane (DMTS; TCI) are useful as observation solvents for single-molecule imaging of a polymer. Because the interaction between the polymer chain and substrate depends on the observation solvent, a solvent suitable for observing the dynamic state of the polymer chain was selected. The structural dynamics of the unmodified SBR polymer were observed at 25 ± 1 °C in DMTS by fast-scanning (high-speed) AFM after adsorption on a mica substrate (Movie S1&2).

Here, the hydrophilic silica microparticle surface can be simulated by a mica substrate, because both have hydroxyl groups (as silanol) on their surface. The densities of surface hydroxyl groups of mica (muscovite) and hydrophilic silica microparticles were 3.2 and 4.7 per $nm^2$, respectively.

In the analysis of dynamics in a polymer chain, each measurement point of the video-imaged polymer chain was tracked and the mean square displacement (MSD) for a certain time Δ*t* was plotted against Δ*t*,

$$MSD(\Delta t) = \overline{[\Delta x(\Delta t)]^2 + [\Delta y(\Delta t)]^2} \, .$$

The diffusion coefficient *D* ($nm^2/s$) was calculated by dividing the slope of the linearly approximating the MSD-Δ*t* plots of each measurement point by four. These tracking points were determined by the numbering at equal intervals along the single chain trunk at each frame of the AFM movie.

**Molecular modeling by all-atom MD**

All-atom molecular dynamics (MD) simulations were carried out using the Forcite module of the BIOVIA Materials Studio 2017 (Dassault Systèmes BIOVIA, San Diego, CA, USA) on the supercomputer system (PRIMERGY CX250, Fujitsu, Tokyo, Japan). A 100-mer model of carboxyl-functionalized SBR was build by use of the Polymer Builder module. The copolymerization ratio was based on the data in Chart S1. The MD cell was built by means of usual procedure of the Amorphous Cell module. The MD cell length and angle were (a = 200 Å, b = 100 Å, c = 150 Å) and (α = 90º, β = 90º, γ = 90º), respectively. Here, single polymer chain was put in the center of the cell, and the solvent molecules of *n*-octylbenzene were packed in the cell at density of 0.858 g $cm^{-3}$. Sequentially, the geometry of the MD cell was optimized. Simulation in the NVT ensemble (constant number of atoms, volume and temperature) was conducted at 298 K for 20 ps (time step of 0.2-fs, 100,000 steps) and the NPT ensemble (constant number of atoms, pressure and temperature) was conducted at pressure of $1.013 \times 10^{-4}$ GPa and at 298 K for 80 ps (time step of 1.0-fs, 80,000 steps) to equilibrate the MD cell. The Nose thermostat was used to control the temperature. The Berendsen barostat was used to control the pressure. After the equilibration at 298 K, simulation in the NVT ensemble (constant number of atoms, volume and temperature) was conducted at 600 K for 20 ps (time step of 1.0-fs, 20,000 steps) as the thermal equilibration. After the equilibration at 600 K, simulation in the NVE ensemble (constant number of atoms, volume and energy) was conducted for 6.5 ns (time step of 1.0-fs, 6,500,000 steps) as the production run. The COMPASS II (ver. 1.2) forcefield was used, and the charges were assigned by the forcefield. A snapshot structure was indicated in Fig. S7.

**Results and discussion**

**Spring constant of a single polymer chain**

The relationship between the energy stored by a spring ($E$) and thermal energy ($k_B T$) can be described as follows:

$$E = \frac{1}{2} k_{chain} x^2 = k_B T .$$

Here, $k_{chain}$ is the spring constant, and $x$ is the displacement of the spring. The temperature $T$ was 298 K, and the Boltzmann constant is $k_B$ =1.38×10$^{-23}$ J/K (J = N·m). Thus,

$$k_B T = 4.11 \times 10^{-21} \text{ [N·m]}.$$

**(1) Unmodified SBR.** Based on the 100-points measurement of the trajectories along a polymer chain (Movie S4), the average length of an unmodified SBR polymer chain was 140 nm with SD = 17.2 nm ($n$ = 30) in DMTS as an observation solvent. The $x$ of the spring of the unmodified SBR chain is its SD; i.e.,

$$x = 17.2 \times 10^{-9} \text{ [m]}.$$

A single unmodified SBR chain therefore has a $k_{chain}$ of

$$k_{chain} = \frac{2 \times k_B T}{x^2} = \frac{2 \times 4.11 \times 10^{-21}}{(17.2 \times 10^{-9})^2} = 2.78 \times 10^{-5} \text{ [N/m]}$$

$$= 2.78 \times 10^{-2} \text{ [pN/nm]}.$$

In terms of molecular stiffness, this value is close to the soft stiffness of a biomacromolecular chain of the myosin subfragment 2 of single myosin molecules in myofilaments.[8]

In addition, the force at $x$ = 17.2 nm is,

$$F = k_{chain} x = 2.78 \times 10^{-2} \times 17.2$$

$$= 4.78 \times 10^{-1} \text{ [pN]}.$$

Compared with the force of an unmodified SBR chain, the force of the carboxyl-functionalized SBR chain is 6.07 times larger (see the next part 2). Thus, the effect of the introduction of the modifying group could be evaluated by mechanical parameters such as spring constant and force.

**(2) Carboxyl-functionalized SBR (SBR-(COOH)-).** The carboxyl-functionalized SBR in *n*-octylbenzene as an observation solvent had four anchor points in a single polymer chain (Fig. 3A). Based on the 100-points measurement of the trajectories along a polymer chain (Movie S6), the average length of a functionalized SBR chain was 142 nm with SD = 2.84 nm ($n$ = 31). The $x$ of the spring of the functionalized SBR chain is its SD; i.e.,

$$x = 2.84 \times 10^{-9} \text{ [m]}.$$

The spring constant of the carboxy-functionalized SBR chain was calculated as

$$k_{chain} = \frac{2 \times k_B T}{x^2} = \frac{2 \times 4.11 \times 10^{-21}}{(2.84 \times 10^{-9})^2} = 1.02 \times 10^{-3} \text{ [N/m]},$$

$$= 1.02 \text{ [pN/nm]}.$$

This value is 36.7 times higher than the spring constant of the unmodified SBR chain in the observation solvent DMTS (see the previous part 1).

In addition, the force at $x$ = 2.84 nm is,

$$F = k_{chain} x = 1.02 \times 2.84 = 2.90 \text{ [pN]}.$$

Furthermore, the spring constant and the force of each segment between the four anchor points (a, b, c, and d, see Fig. 4A) were also calculated.

The spring constant $k_{ab}$, $k_{bc}$, and $k_{cd}$ were 6.55, 11.0, and 8.30 [pN/nm], respectively. For example, spring constant between anchor points a and b was indicated as $k_{ab}$.

Here, the displacement of the spring $x_{ab}$, $x_{bc}$, and $x_{cd}$ were 1.12, 0.866, and 0.995 [nm]. Chart S3 shows the classical spring model.

**Chart S3.**

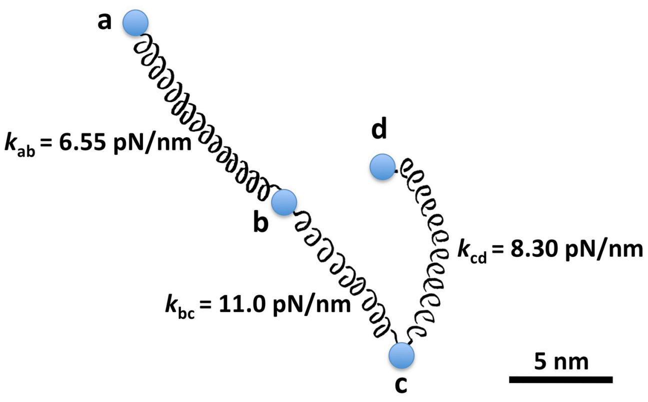


In addition, the force $F_{ab}$, $F_{bc}$, and $F_{cd}$ were 7.34, 9.53, and 8.26 [pN], respectively.

This is the first calculation of the spring constant and force of a single SBR polymer chain.

**Relationships between tire performance and single polymer chains**

The macromolecular movement of the functionalized SBR in an organic medium on mica is considered to resemble that of an actual functionalized SBR with silica in a tire. Therefore, observing and quantifying such behavior should reveal the factors affecting tire performance, such as energy loss and wet grip, at a molecular level. The wet-grip performance (tan δ at 0 °C) of a vulcanized silica-blended functionalized SBR was 1.4 times higher than that of a silica-blended unmodified SBR.[2] This result strongly correlates with *D* of a single polymer chain (Table S1). The *D* values of a single chain of carboxyl-functionalized SBR ranged from 0.03 to 5.97 $nm^2/s$ (Fig. S6), while those of an unmodified SBR were from 0.09 to 33.2 $nm^2/s$ (Fig. 2D). Because the *D* values of carboxyl-functionalized SBR were low and within a narrow range, its glass transition temperature ($T_g$) was higher than that of the unmodified SBR. This result indicates that the macromolecular motion of carboxyl-functionalized SBR was strongly controlled by the interaction between the functional groups and mica (or silica) in an organic medium at 25 °C. In addition, the controlled polymer chain motion of the functionalized SBR was measured as an increase in spring constant (*k*; N/m). This is the origin of the high tan δ (maximum) of silica-blended functionalized SBR. These measured values for single polymer chains on mica in an organic medium correlate with $T_g$ values (as the temperature at maximum tan δ) of SBRs in silica-blended compounds. Actually, $T_g$ of a silica-blended oil-extended rubber containing carboxyl-functionalized SBR was –14 °C, and $T_g$ of that with unmodified SBR was –20 °C.[2]

The rolling resistance of tire performance (tan δ at 60 °C) of a vulcanized compound of a silica-blended rubber containing carboxyl-functionalized SBR was smaller than that of silica-blended rubber of unmodified SBR.[2] This result also strongly correlates with *D* of a single polymer chain (Table S1). The *D* values ($D_2$-$D_{18}$) of carboxyl-functionalized SBR were lower than those of unmodified SBR; that is, by introducing the functional groups into the SBR polymer chain, excessive movement in the polymer chain containing the chain end was moderately suppressed.

**Data:**

**Single chain of unmodified SBR**

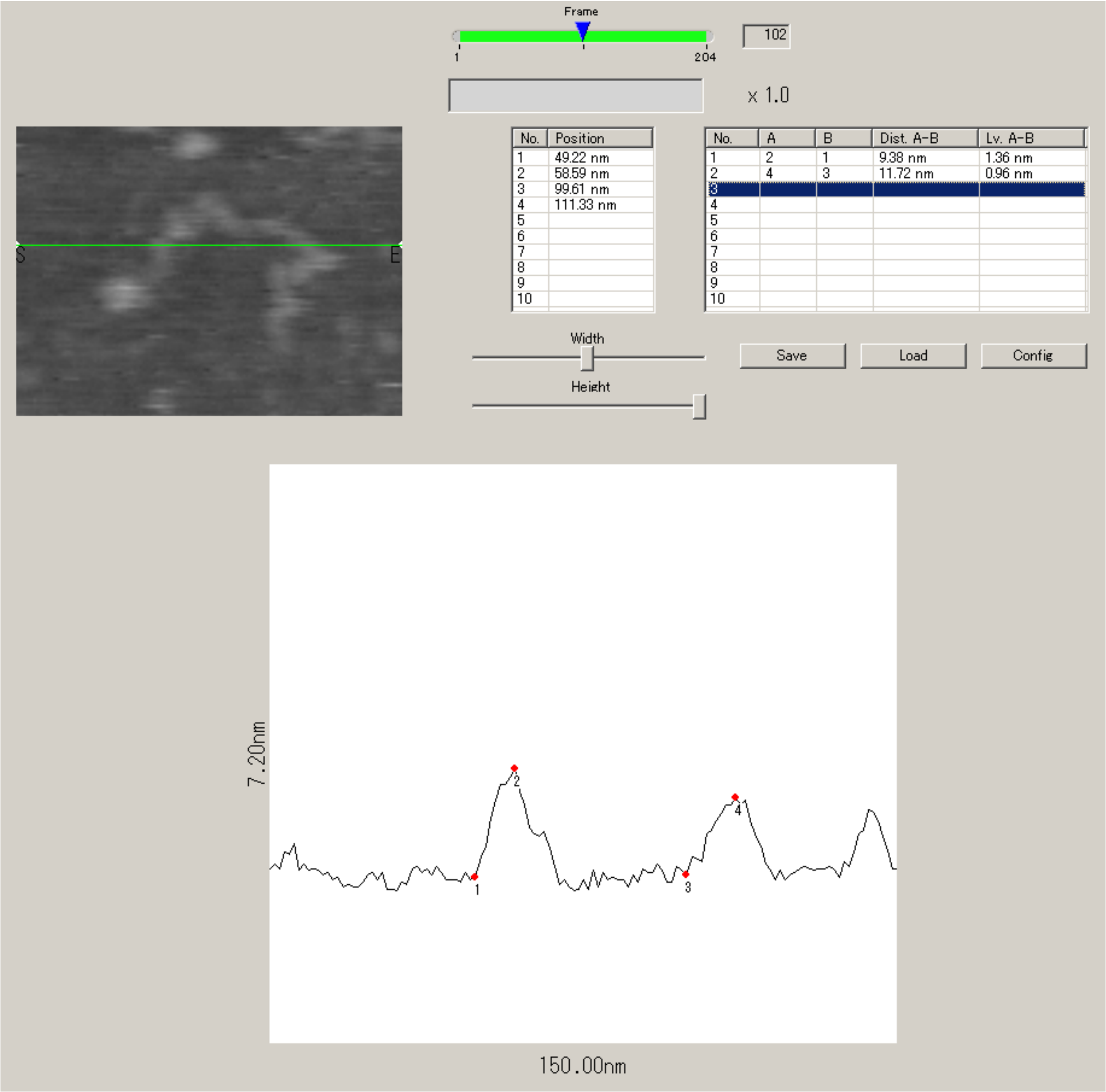


**Fig. S1A.** Line profile of AFM image of single polymer chain of unmodified SBR (see Fig. 2).

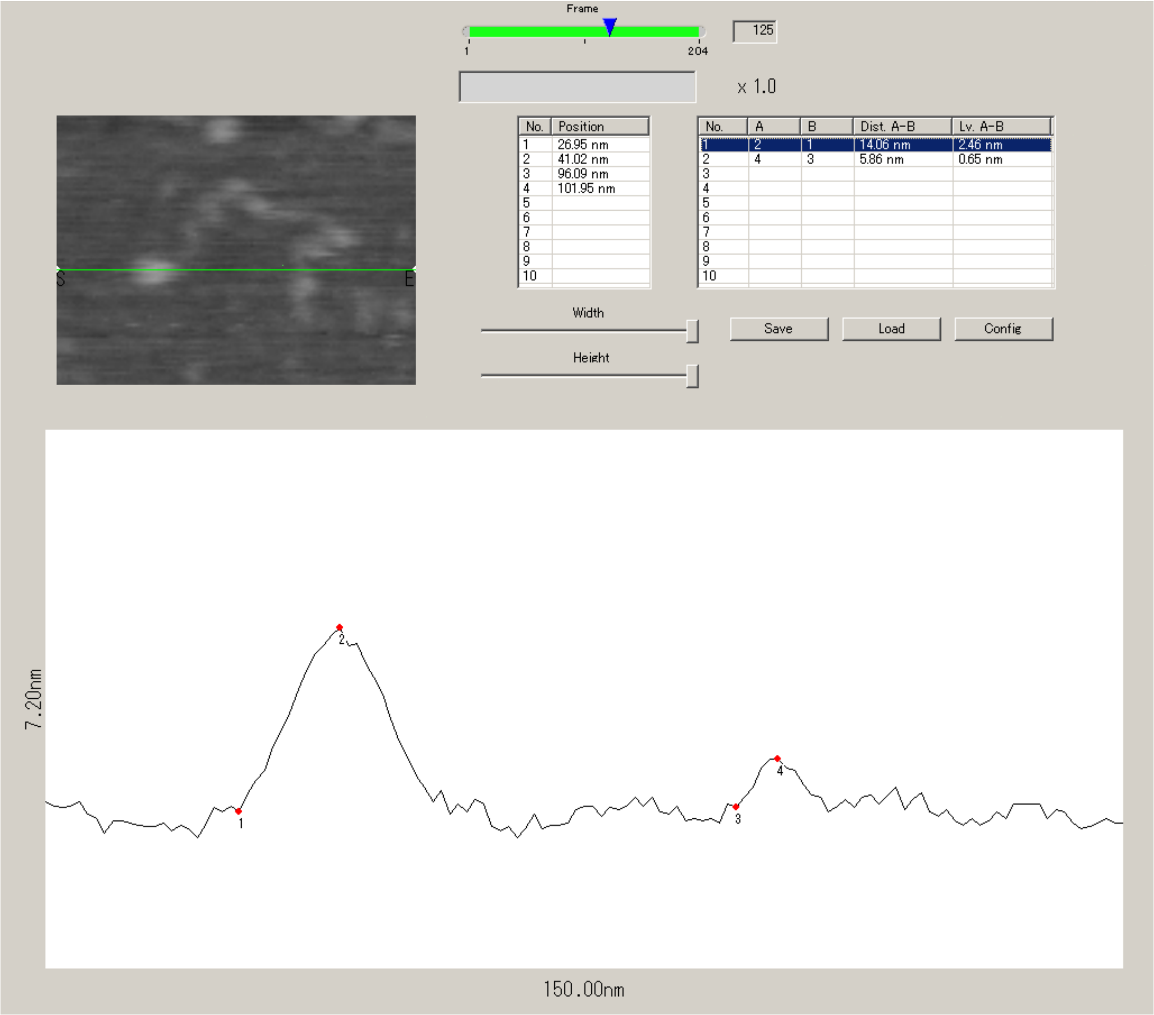


**Fig. S1B.** Line profile of AFM image of single polymer chain of unmodified SBR (see Fig. 2).

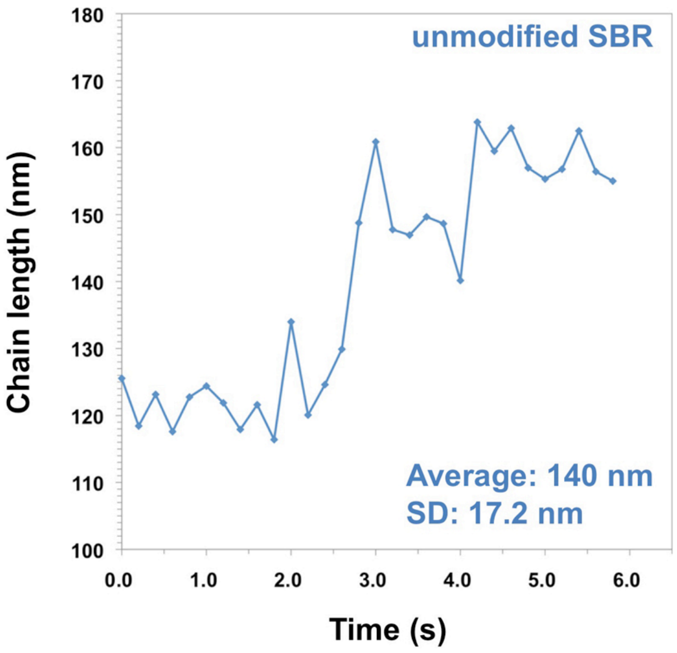


**Fig. S2.** Chain length-time plots of an unmodified SBR (see Movie S4).

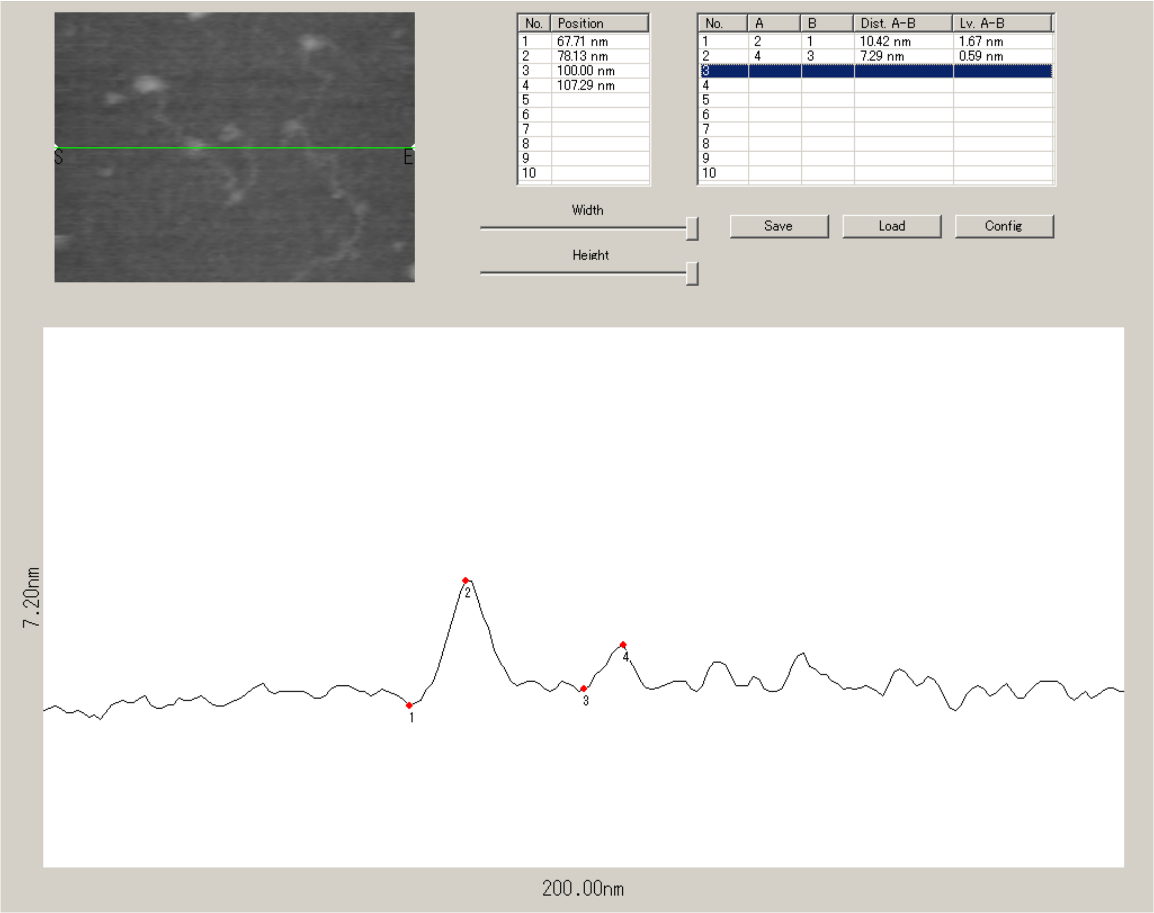


**Fig. S3.** Line profile of AFM image of single polymer chain of a carboxyl-functionalized SBR (see Fig. 3 and Movie S5).

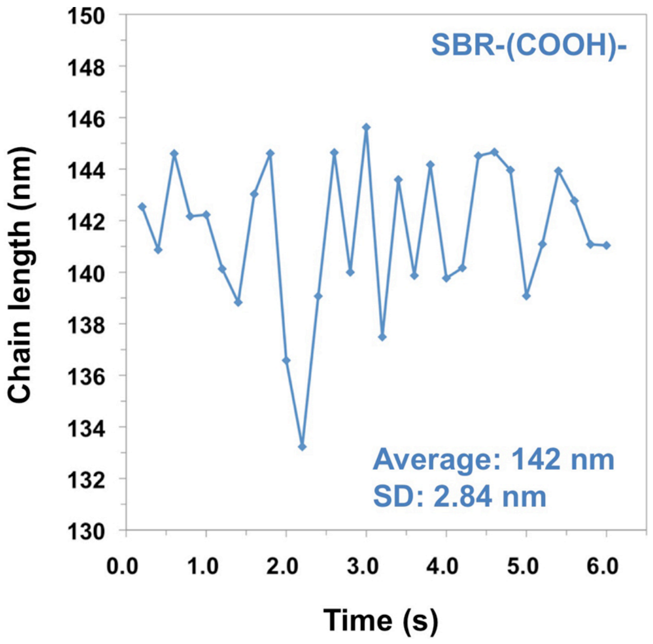


**Fig. S4.** Chain length-time plots of a carboxyl-modified SBR (see Movie S6).

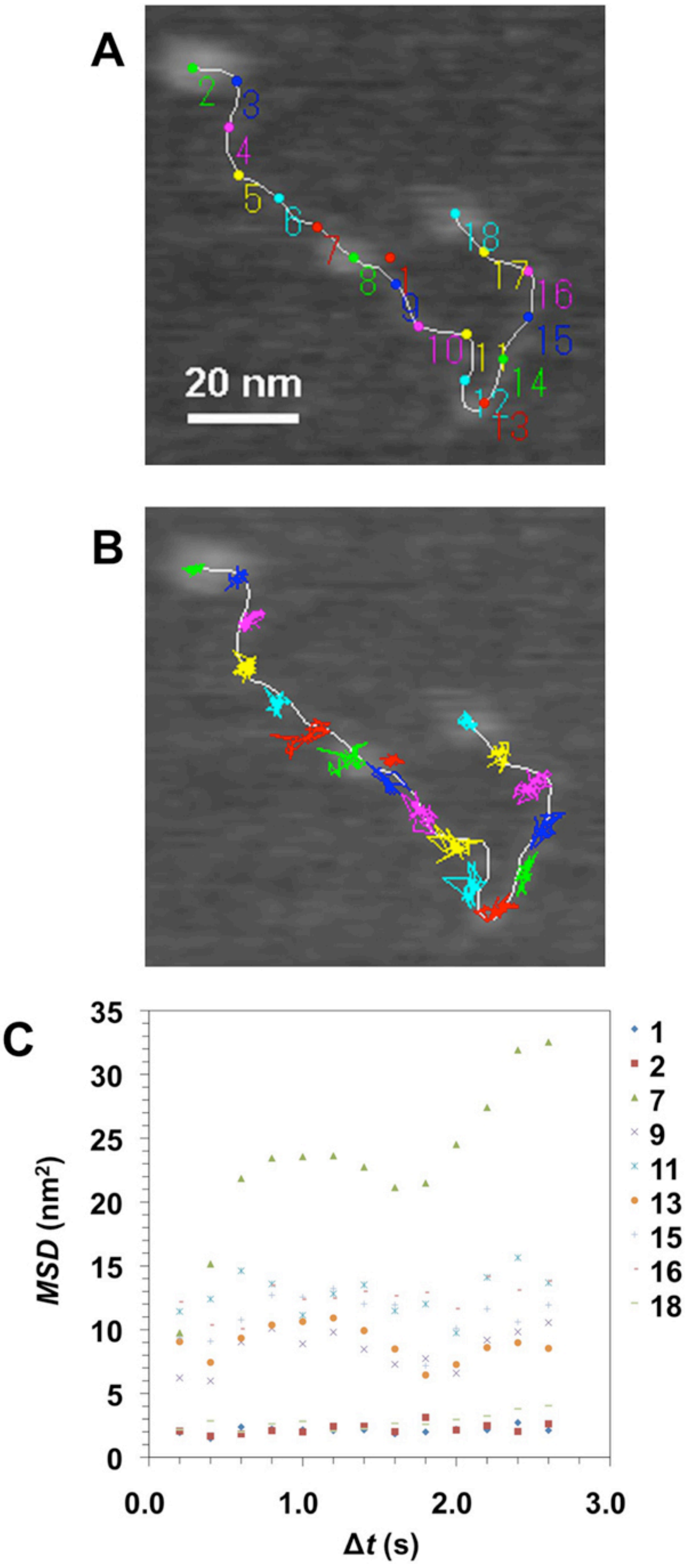


**Fig. S5.** Dynamic structural analysis of a carboxyl-functionalized SBR on mica under *n*-octylbenzene at 25 ± 1 °C. (A) The 18-points measurement points were indicated over an AFM snapshot. (B) The trajectories. (C) Mean square displacement (MSD)-Δ*t* plots in the single polymer chain

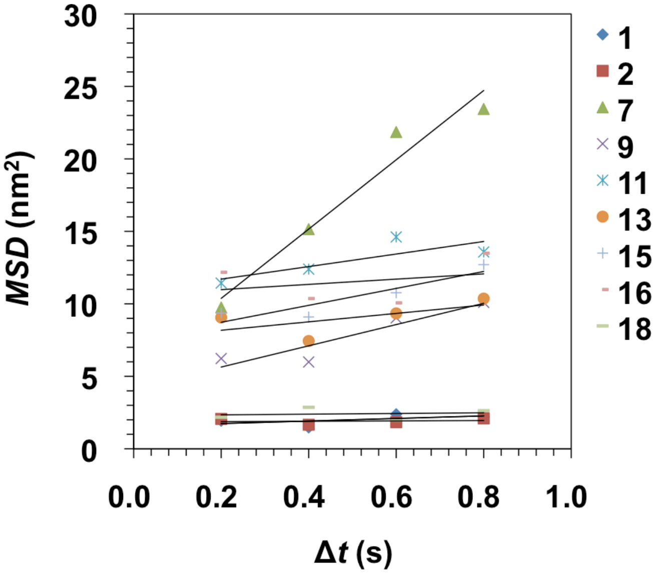


**Fig. S6.** Mean square displacement (MSD)-Δ*t* plots (Δ*t* ≤ 0.8 s from Fig. S5C) of 18-points measurement in single polymer chain of a carboxyl-functionalized SBR for calculation of the *D* values as shown in Table S1

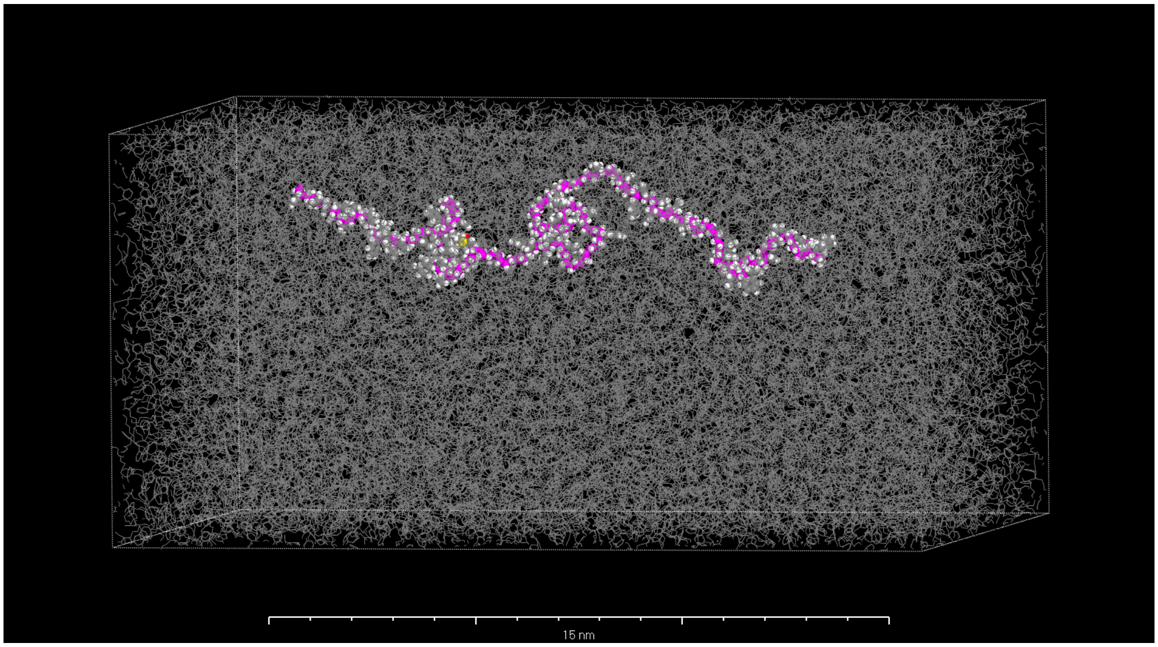


**Fig. S7.** All-atom molecular dynamics (MD) simulated structure of a 100-mer carboxyl SBR chain in *n*-octylbenzene by the NVE ensemble after the equilibration calculation at 600 K. This MD snapshot is at 6.44 ns. The backbone was displayed in purple. The copolymerization ratio was based on the data in Chart S1. Scale bar: 15 nm. Dynamic small globular structures were confirmed in a chain.

**Table S1.** Diffusion coefficients[a)] of single polymer chains of a carboxyl-functionalized styrene-butadiene rubber (SBR) [SBR-(COOH)-] and unmodified SBR in organic solvents on mica at 25 ± 1 °C.

| Four fixing points in a chain of SBR-(COOH)- | | A chain of SBR-(COOH)- | | A chain of unmodified SBR | |
|---|---|---|---|---|---|
| in *n*-octylbenzene, ΔSP = 0.1 [b)] ($SP_{SBR} - SP_{n\text{-octylbenzene}}$) | | | | in DMTS, ΔSP = 3.9 [b)] ($SP_{SBR} - SP_{DMTS}$) | |
| $D_a$ | 0.07 | $D_1$ | 0.22 [c)] | $D_1$ | 3.83 [c)] |
| $D_b$ | 0.13 | $D_2$ | 0.03 | $D_2$ | 0.09 |
| $D_c$ | 0.07 | $D_7$ | 5.97 | $D_6$ | 2.17 |
| $D_d$ | 0.11 | $D_9$ | 1.83 | $D_8$ | 6.82 |
| | | $D_{11}$ | 1.08 | $D_{10}$ | 13.4 |
| | | $D_{13}$ | 0.73 | $D_{12}$ | 29.2 |
| | | $D_{15}$ | 1.46 | $D_{13}$ | 33.2 |
| | | $D_{16}$ | 0.45 | $D_{16}$ | 1.47 |
| | | $D_{18}$ | 0.06 | $D_{18}$ | 3.15 |

a) Unit: $nm^2/s$. b) Difference of two solubility parameters (ΔSP). Compared with DMTS, *n*-octylbenzene has high solubility for SBR. c) A centroid of a polymer chain.

**Supplementary References**

1. T. A. Antkowiak, A. E. Oberster, A. F. Halasa and D. P. Tate, *J. Polym. Sci. Part A-1: Polym. Chem*., 1972, **10**, 1319.

2. N. Steinhauser and A. Lucassen, paper presented at the BPRI Conference, Brussels, Belgium, 25 November 2009. https://ja.scribd.com/document/69961410/SSBR

3. Bayer Aktiengesellschaft, Pat ., EP 1 000 971 A1, 2000.

4. K. Shinohara, Jpn. Pat., P5907484, 2016.

5. K. Shinohara, Pat., WO 2014104172 A1, 2014.

6. T. Ando, N. Kodera, E. Takai, D. Maruyama, K. Saito and A. Toda, Proc. Natl. Acad. Sci. USA, 2001, **98,** 12468.

7. T. Ando, Nanotechnology, 2012, 23, 062001.

8. M. Kaya and H. Higuchi, *Science*, 2010, **329**, 686.
